# Frequency‐Dependent Effects of Warm Needle Acupuncture in Patients With Knee Osteoarthritis With Cold‐Dampness Obstruction Syndrome: A Randomized Controlled Trial

**DOI:** 10.1155/prm/1674261

**Published:** 2026-10-07

**Authors:** Hanzhi Deng, Qin Su, Yuqing Wang, Jiqing Wang, Kai Liu, Liying Fu, Shanshan Li, Shifen Xu, Yiqun Mi

**Affiliations:** ^1^ Shanghai Municipal Hospital of Traditional Chinese Medicine Shanghai University of Traditional Chinese Medicine Shanghai 200071 China shutcm.edu.cn

**Keywords:** complementary and alternative medicine, knee osteoarthritis, randomized controlled trial, treatment frequency, warm acupuncture

## Abstract

**Background:**

Knee osteoarthritis (KOA) is a common chronic degenerative joint disease. Its rising incidence, driven by population aging and increasing obesity rates, imposes a growing socioeconomic burden. As a therapy integrating the dual merits of acupuncture regulation and thermal stimulation, warm acupuncture has been widely adopted for KOA, accompanied by a growing body of relevant research. However, standardized recommendations regarding optimal treatment frequency and course duration of warm needle acupuncture for KOA remain lacking.

**Methods:**

A total of 84 eligible KOA patients were enrolled and randomly assigned to either a high‐frequency group or a low‐frequency group. The high‐frequency group received 5 treatments per week for the first 2 weeks, whereas the low‐frequency group received 2 treatments per week during the same period. The primary outcome was the between‐group difference in the Western Ontario and McMaster Universities Osteoarthritis Index (WOMAC) pain score at the end of Week 2. Secondary outcomes included the WOMAC stiffness and function scores, active range of motion of the knee joint, medical infrared thermography data, quantitative scoring of traditional Chinese medicine (TCM) syndrome classification, and the Patient Health Questionnaire‐9 (PHQ‐9). Analyses were performed using generalized estimating equations.

**Results:**

The WOMAC pain score in the high‐frequency group after 2 weeks of treatment showed a statistically significant difference compared to the low‐frequency group (difference, 3.07 [95% CI, 1.86 to 4.28], *p* < 0.001). Secondary outcome results indicated that acupuncture, compared to the low‐frequency group, significantly alleviated the WOMAC stiffness score (difference, 0.90 [95% CI, 0.26 to 1.55], *p* = 0.006) and WOMAC functional scores (difference, 6.86 [95% CI, 2.58 to 11.13], *p* = 0.002), as well as active knee range of motion (difference, −11.00 [95% CI, −16.31 to −5.69], *p* < 0.001) and the quantified score for traditional Chinese medicine syndrome grading (difference, 1.71, 95% CI, 0.88 to 2.55, *p* < 0.001). However, no statistically significant differences were observed in the PHQ‐9 total score or knee‐related regional temperature (*p* > 0.05). The incidence of adverse events was similar between the two groups.

**Conclusion:**

Both high‐ and low‐frequency warm acupuncture significantly improved clinical symptoms in patients with KOA. However, high‐frequency warm acupuncture demonstrated markedly superior efficacy compared to low‐frequency treatment in alleviating pain, enhancing joint function, increasing active knee range of motion, and improving TCM syndromes. These findings provide evidence‐based support for optimizing treatment frequency in warm needle acupuncture as a safe and effective non‐pharmacological intervention for KOA.

**Trial Registration:** International Traditional Medicine Clinical Trial Registry: ITMCTR2024000269

## 1. Introduction

Knee osteoarthritis (KOA) is a chronic osteoarticular disease characterized primarily by degenerative changes in articular cartilage and secondary bone proliferation, leading to joint pain, deformity, and restricted mobility [1]. Statistics indicate that KOA affects approximately 360 million people worldwide, with both prevalence and incidence rates rising significantly across most countries and regions over the past three decades [2]. Disability‐adjusted life years (DALYs) lost due to hip and knee osteoarthritis rank first globally. As the disease progresses, worsening symptoms such as joint deformity, limited mobility, and muscle atrophy increasingly become the primary causes of patients’ loss of labor capacity. Prolonged, recurrent pain stimuli also trigger a series of issues affecting daily life quality, including depression and insomnia [3–5]. Current recommended interventions for KOA primarily involve pharmacological approaches, including nonsteroidal anti‐inflammatory drugs (NSAIDs), opioids, and chondroprotectants. However, these carry adverse effects such as increased cardiovascular risk [6] and gastrointestinal mucosal damage [7]. Intra‐articular drug injections offer only transient and limited efficacy [8], while surgical interventions are constrained by high medical costs, prolonged rehabilitation periods, and poor compliance with exercise therapy. The high prevalence and severity of KOA, coupled with the limitations of conventional treatments, make the exploration of superior KOA management strategies an urgent priority.

Warm acupuncture is widely applied in KOA treatment with proven efficacy. Most previous trials focused on observing the clinical efficacy and mechanisms of warm acupuncture combined with other therapies. Ya et al. [9] conducted a randomized controlled trial on 70 KOA patients, demonstrating that warm acupuncture combined with exercise rehabilitation significantly alleviated knee pain, improved knee function, and facilitated the restoration of musculoskeletal balance. However, few studies have addressed the optimization of key parameters such as acupuncture frequency and dosage. Against the current trend in clinical medicine toward consensus and standardization, establishing uniform and appropriate acupuncture parameters is crucial for achieving standardized diagnosis and treatment and enhancing therapeutic efficacy [10]. A literature review indicates [11] that warm acupuncture administered once daily (five times weekly) represents the most common clinical treatment frequency. Our research group’s prior study [12] demonstrated that twice‐weekly warm acupuncture also yielded therapeutic benefits.

Therefore, we conducted this randomized controlled trial to investigate the impact of treatment frequency on KOA outcomes. This research is crucial for optimizing treatment protocols, enhancing therapeutic efficacy, and reducing time and economic costs, aiming to fill the research gap in the dose–response relationship of warm acupuncture for KOA. Supporting Information eFigure 1 shows the graphical abstract summarizing the study design, intervention protocols, and main findings.

## 2. Study Design

This randomized clinical trial was conducted from September 7, 2024, to October 31, 2025, at the Acupuncture Department and Orthopedics Department of Shanghai Hospital of Traditional Chinese Medicine. The entire trial comprised three phases: the first phase was a two‐week treatment period, with the high‐frequency group receiving treatment five times weekly (five consecutive days followed by two days of rest) and the low‐frequency group receiving treatment twice weekly (with a three‐day interval between each session). The second phase was a four‐week maintenance period with both groups receiving one treatment session per week. The third phase was a 12‐week posttreatment follow‐up period. The study protocol was approved by the Ethics Review Committee of Shanghai Hospital of Traditional Chinese Medicine (ID: 2024SHL‐KY‐80‐02). The trial was conducted in accordance with the principles of the Declaration of Helsinki. Additionally, the study was registered with the China Clinical Trial Registry This study adheres to the Consolidated Standards of Reporting Trials (CONSORT) guidelines and the Standards for Reporting Interventions in Clinical Trials of Acupuncture (STRICTA) guidelines.

## 3. Subjects

Recruitment strategies primarily included posting recruitment advertisements on the Shanghai Hospital of Traditional Chinese Medicine’s WeChat public account and offering free medical consultations in outpatient departments. Participants meeting all the following criteria were included: (1) meeting the Western medical diagnostic criteria for KOA as outlined in the “Diagnosis and Treatment Guidelines for Osteoarthritis (2018 Edition) [13]” and the traditional Chinese medicine (TCM) pattern differentiation criteria for KOA as “Cold‐Dampness Bi Syndrome” according to the “Diagnosis and Treatment Guidelines for Osteoarthritis Combining Pattern Differentiation [14]”; (2) aged 50–80 years, regardless of gender; (3) knee radiographic Kellgren–Lawrence (KL) Grade I–III; (4) alert and oriented, able to understand questionnaire items, and complete assessments; (5) willingness to participate in the trial and provide signed written informed consent. Prior to randomization, comprehensive evaluations including physical examination, routine laboratory tests, and radiographic studies were conducted to exclude participants with underlying organic pathology. Participants meeting any of the following criteria were excluded: (1) a history of knee arthroplasty, significant acute trauma, or concomitant knee tumors, tuberculosis, or osteomyelitis; (2) had arthroscopy within the past 6 months or intra‐articular injections of steroids or hyaluronic acid within the past 3 months; (3) were medically or psychologically unfit for treatment as assessed by a physician, including severe acute or chronic organic diseases of the heart, liver, kidneys, cerebrovascular system, or hematopoietic system; recent stroke; active cancer; psychiatric disorders; cognitive impairment; or communication barriers; or (4) had enrolled in another clinical trial within the past month. All participants provided written informed consent.

## 4. Randomization and Blinding

This trial employed a block randomization method for grouping, conducted by an independent statistical expert. Subjects were assigned in a 1:1 ratio to either the high‐frequency group or the low‐frequency group. The block size was 4, with 2 groups. Randomized number tables were generated using SPSS 26.0 software. Randomization concealment was achieved via random envelopes—opaque, sealed duplicates delivered to the study sponsor and research center respectively. Cases were assigned according to the envelope instructions: 42 patients in the high‐frequency group and 42 in the low‐frequency group, totaling 84 participants. Due to the nature of the warm needle acupuncture intervention, blinding of patients and acupuncturists was not feasible. To minimize subjective bias, patients were assigned to separate treatment rooms, with each patient isolated in a closed ward. All study personnel underwent multiple rounds of standardized training to clarify key quality control points. The “separation of duties principle” was strictly adhered to throughout the trial: personnel responsible for the intervention did not participate in efficacy assessment, while those handling data collection and statistical analysis had no contact with the intervention process.

## 5. Intervention

### 5.1. Treatment Phase and Maintenance Phase

The treatment phase defined the period during which the high‐frequency and low‐frequency groups received therapy at their respective frequencies: the high‐frequency group received treatment 5 times per week, and the low‐frequency group received treatment 2 times per week, for a total of 2 weeks.

The maintenance phase was defined as the period following the treatment phase, during which both groups uniformly received weekly treatments to consolidate efficacy, lasting 4 weeks.

### 5.2. Grouping

#### 5.2.1. High‐Frequency Group

Patients received warm needle acupuncture treatment. Patients assumed a supine or seated position with a rolled towel placed under the affected knee to maintain natural knee flexion. After skin disinfection, acupuncture points on the affected side included Dubi (ST35), Neixiyan (EX‐LE4), Yanglingquan (GB34), Yinlingquan (SP9), Liangqiu (ST34), Xuehai (SP10), and Heding (EX‐LE2) on the affected side. The flat tonification and flat dispersion technique was applied, with needles retained after deqi. Moxa cones were placed over the needle shafts at ST35 and EX‐LE4. A distance of approximately 1.5 cm was maintained between the moxa cone and the skin. The moxa cone was ignited from its base. Each cone was burned for 10 min, with two consecutive cones used per treatment. The needles were left in place for 20 min before removal.

#### 5.2.2. Low‐Frequency Group

Acupoint selection and manipulation methods are identical to those in the high‐frequency group, but the treatment frequency differs.

### 5.3. Acupuncturist Qualifications

Practitioners must hold a valid physician’s license with at least 5 years of clinical acupuncture experience. All acupuncturists will undergo standardized training in acupoint localization and warm moxibustion techniques prior to study initiation.

Supporting Information eTable 1 details acupoint locations and associated meridians, while eTable 2 provides further intervention specifications. See eFigure 2 for the acupoint diagram.

## 6. Outcome Measures

The Western Ontario and McMaster Universities Osteoarthritis Index (WOMAC) and Patient Health Questionnaire‐9 (PHQ‐9) health questionnaires were administered at Week 0 (baseline), Week 2 (during treatment), Week 6 (posttreatment), and Week 12 (follow‐up). Knee active range of motion (AROM), medical infrared thermography, and TCM syndrome grading quantitative scoring were assessed at Week 0 (baseline), Week 2 (during treatment), and Week 6 (posttreatment). eTable 3 outlines the study timeline.

The primary outcome measure was the between‐group difference in the WOMAC pain score at Week 2. WOMAC is a widely used self‐report questionnaire for KOA globally, comprising 24 items scored 0–4 points each. It includes 5 knee pain items (0–20 points), 2 knee stiffness items (0–8 points), and 17 daily activity items (0–68 points), yielding a total score of 0–96 points. Higher scores indicate more severe osteoarthritis.

Secondary outcome measures included the WOMAC stiffness score, WOMAC function score, AROM, medical infrared thermography, TCM syndrome grading quantitative scoring system, and PHQ‐9 health questionnaire. AROM assessed flexion/extension function. Patients sat with knees flexed at 90°, then actively flexed/extended until pain onset. A protractor measured range of motion, averaging three measurements for accuracy. Medical infrared thermography monitored temperature changes in knee‐related regions using the Xinhang Medical Infrared Thermography Instrument TMT‐9000P (Hangzhou Xinhang Optoelectronics Technology Co., Ltd.). Examinations were conducted in a room with minimal air convection, maintained at (25 ± 1)°C and (50 ± 10)% humidity. Patients were required to sit quietly for 15 min in this environment with the exposed examination area to acclimate to room temperature and stabilize their emotions. Subsequently, they stood in front of the infrared camera in a standard anatomical posture. Temperatures were measured and recorded at five observable points on the skin surface before and after treatment: the medial tibial plateau, lateral tibial plateau, medial femoral condyle, lateral femoral condyle, and suprapatellar region. Additionally, the TCM Syndrome Grading Quantification Score Sheet was used to assess six dimensions: joint pain, swelling, aversion to cold, fever, weakness in the lower back and knees, and fatigue. Each dimension was scored on a 0–3 scale, yielding a total score range of 0–18. Higher scores indicate more severe corresponding TCM syndrome symptoms. The PHQ‐9 health questionnaire was also used to assess depressive disorders. This questionnaire comprises 9 items, each scored 0–3 points, with a total score range of 0–27 points. Higher scores indicate more severe depression.

Adverse reactions potentially related to the intervention, such as burns, subcutaneous hematoma, skin itching, and worsening knee pain, were recorded during the trial.

## 7. Statistical Analysis

The minimum sample size of 84 (42 per group) was determined based on prior studies and Wang Xiaoling’s [15] research findings. The expected difference in WOMAC pain score change between the high‐frequency and low‐frequency groups was 2 points, with a standard deviation of 2.5 points for both groups. We set the significance level at *α* = 0.05 and the power at 1‐β = 0.9 Substituting these values into the sample size formula for comparing two independent means yields a sample size of 33 per group. Accounting for a 20% dropout rate, each group required 42 subjects, totaling 84 subjects. Data analysis was performed by an independent statistician unaware of group assignments using SPSS 28.0 statistical software. All cases in this study were analyzed using the intention‐to‐treat (ITT) population, including data from subjects who withdrew during the trial period. Missing data postwithdrawal will be imputed using the most recent available case data for each subject.

Descriptive analysis was used for baseline characteristics of each patient group. Primary outcomes and all secondary outcomes were assessed using generalized estimating equations (GEEs) with a Gaussian family, identity link function, and exchangeable working correlation structure, which specifies a linear model for the marginal mean response, with the patient as the clustering variable to account for within‐subject correlation over repeated measurements. The model included treatment group, time, and the treatment‐by‐time interaction as primary independent variables and was adjusted for the following prespecified baseline covariates: gender, age, side, disease duration, BMI, KL classification, and baseline score. Robust (sandwich) standard errors were used. *p* < 0.05 was considered statistically significant, and all statistical tests were two‐tailed.

## 8. Results

### 8.1. Baseline Characteristics

A total of 146 patients with KOA were screened, of whom 84 met the eligibility criteria and were randomly assigned in a 1:1 ratio to the high‐frequency group or the low‐frequency group. The study population included 73 females (86.9%) and 11 males (13.1%), with a mean (SD) age of 65.02 ± 8.34 years. Follow‐up was completed for 80 subjects throughout the trial; four participants withdrew from the trial. Two participants in the high‐frequency group dropped out due to long commuting distances. The other two participants belonged to the low‐frequency acupuncture group: one withdrew owing to job relocation, and the other was transferred to the Department of Endocrinology for treatment because of disease progression (Figure 1). Baseline demographic characteristics and clinical parameters were comparable between the two groups, with no statistically significant differences observed, indicating good baseline balance (Table 1).

**FIGURE 1:**
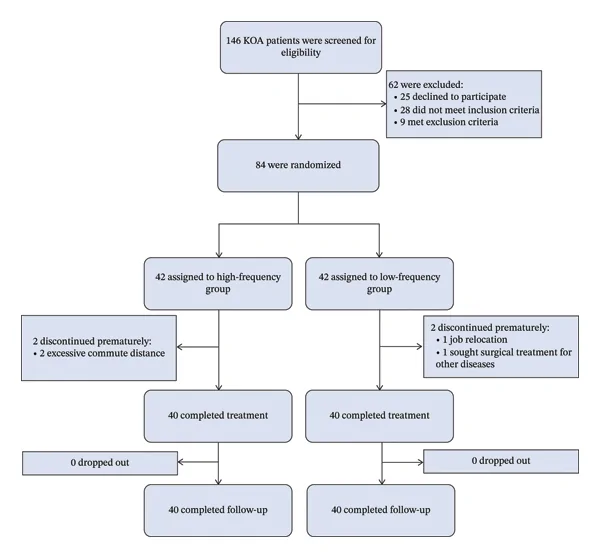
Research flowchart.

**TABLE 1:** Baseline demographic and clinical characteristics.

Characteristics	Study group	
	HF (*n* = 42)	LF (*n* = 42)
Gender (%)		
Male	6 (14%)	5 (12%)
Female	36 (86%)	37 (88%)
Age (years)	66.21 ± 8.65	63.83 ± 7.94
Side		
Left	21 (50)	18 (43)
Right	21 (50)	24 (57)
Duration, months	49.5 (16.75, 160)	78.5 (14.75, 121)
BMI (kg/m^2^)	23.85 ± 3.24	24.18 ± 2.88
Prior acupuncture treatment (%)		
Yes	24 (57%)	20 (48%)
No	18 (43%)	22 (52%)
KL classification		
I	17	15
II	14	15
III	11	12
WOMAC pain score	8.76 (7.95, 9.57)	8.67 (7.73, 9.61)
WOMAC stiffness score	3.21 (2.77, 3.65)	3.05 (2.59, 3.50)
WOMAC function score	29.83 (26.88, 32.78)	27.24 (24.45, 30.03)
Knee active range of motion	72.19 (67.83, 76.55)	72.93 (68.48, 77.38)
Medial tibial plateau temperature	33.87 (33.42, 34.31)	33.71 (33.33, 34.09)
Lateral tibial plateau temperature	34.18 (33.78, 34.57)	33.88 (33.59, 34.19)
Medial femoral condyle temperature	33.96 (33.53, 34.39)	33.78 (33.39, 34.18)
Lateral femoral condyle temperature	33.33 (32.89, 33.77)	33.02 (32.64, 33.39)
Supracondylar temperature	33.63 (33.20, 34.05)	33.45 (33.04,33.85)
Traditional Chinese medicine syndrome grading and quantitative scoring	8.71 (8.19, 9.24)	8.10 (7.52, 8.67)
PHQ‐9 health questionnaire	4.40 (3.00, 5.80)	4.95 (3.77, 6.14)

### 8.2. Primary Outcome

For the primary outcome measures, a separate effect analysis between groups revealed that the WOMAC pain score at Week 2 was significantly lower in the high‐frequency group than in the low‐frequency group by 3.07 points (95% CI, 1.86 to 4.28; *p* < 0.001) (see Table 2). In terms of descriptive statistics, the mean within‐group change from baseline to Week 2 was 2.88 (95% CI, 2.13 to 3.63) in the high‐frequency group and 5.95 (95% CI, 5.00 to 6.91) in the low‐frequency group (see Table 3). The trend in WOMAC pain score changes from baseline to follow‐up for participants in both groups is shown in Figure 2.

**TABLE 2:** Comparison of primary outcomes between high‐frequency and low‐frequency groups.

Outcome	HF vs. LF	
	Mean difference (95% CI)	*p*
Week 2	3.07 (1.86, 4.28)	< 0.001
Week 6	1.81 (0.70, 2.92)	0.001
Week 12	1.214 (0.10, 2.33)	0.034

**TABLE 3:** Results of primary outcomes for high‐frequency and low‐frequency groups.

Outcome	HF (*n* = 42)	LF (*n* = 42)
	Mean score (95% CI)	
Week 2	2.88 (2.13, 3.63)	5.95 (5.00, 6.91)
Week 6	2.07 (1.37, 2.78)	3.88 (3.03, 4.73)
Week 12	2.62 (1.79, 3.45)	3.83 (3.08, 4.58)

**FIGURE 2:**
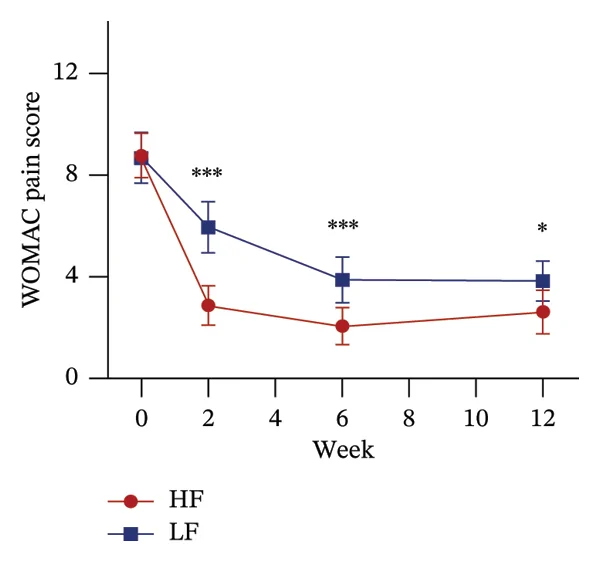
Trend chart of key outcome measures at different time points.

### 8.3. Secondary Outcomes

As shown in Table 4, the high‐frequency group exhibited significantly greater improvements than the low‐frequency group in multiple secondary outcomes. At Week 6, WOMAC stiffness scores were significantly lower in the high‐frequency group (mean difference, 0.90, [95% CI, 0.26 to 1.55], *p* = 0.006), as were WOMAC functional scores (mean difference, 6.86, [95% CI, 2.58 to 11.13], *p* = 0.002). Additionally, participants receiving high‐frequency warm needle acupuncture demonstrated a significantly greater increase in active knee range of motion compared with those in the low‐frequency group (mean difference, −11.00, [95% CI, −16.31 to −5.69], *p* < 0.001). The quantitative TCM syndrome score was also significantly reduced in the high‐frequency group relative to the low‐frequency group at Week 6 (mean difference, 1.71, [95% CI, 0.88 to 2.55], *p* < 0.001). However, no significant differences were observed between groups at Week 6 in PHQ total scores (1.36, [95% CI, −0.20 to 2.91], *p* = 0.09), medial tibial plateau (0.24, [95% CI, −0.44 to 0.91], *p* = 0.49), lateral tibial plateau (0.07, [95% CI, −0.50 to 0.64], *p* = 0.80), medial femoral condyle (0.21, [95% CI, −0.52 to 0.93], *p* = 0.58), lateral femoral condyle (0.08, [95% CI, −0.62 to 0.78], *p* = 0.82), and suprapatellar region (0.25, [95% CI, −0.47 to 0.97], *p* = 0.50). Overall, except for the PHQ‐9 total score and knee‐related regional temperatures, improvement trends in secondary outcome measures largely aligned with the primary outcome (see Figure 3).

**TABLE 4:** Comparison of secondary outcomes between high‐frequency and low‐frequency groups.

Outcomes	HF (*n* = 42)	LF (*n* = 42)	HF vs. LF	
	Mean score (95% CI)		Mean difference (95% CI)	*p*
WOMAC stiffness score				
Week 2	1.38 (0.89, 1.87)	2.24 (1.79, 2.68)	0.86 (0.20, 1.52)	0.011
Week 6	0.93 (0.54, 1.32)	1.83 (1.32, 2.34)	0.90 (0.26, 1.55)	0.006
Week 12	1.12 (0.73, 1.51)	1.57 (1.11, 2.03)	0.45 (−0.15, 1.06)	0.141
WOMAC functional score				
Week 2	10.76 (7.98, 13.54)	18.40 (15.02, 21.79)	7.64 (3.26, 12.03)	0.001
Week 6	8.95 (6.09, 11.82)	15.81 (12.63, 18.99)	6.86 (2.58, 11.13)	0.002
Week 12	10.48 (7.22, 13.73)	14.81 (11.46, 18.16)	4.33 (‐0.34, 9.00)	0.069
AROM				
Week 2	93.02 (88.79, 97.26)	84.17 (79.90, 88.43)	−8.86 (−14.87 to −2.85)	0.004
Week 6	99.81 (96.57, 103.05)	88.81 (84.60, 93.02)	−11.00 (−16.31 to −5.69)	< 0.001
Medial tibial plateau temperature				
Week 2	33.15 (32.65, 33.63)	33.51 (33.10, 33.91)	0.36 (−0.28, 1.00)	0.28
Week 6	33.12 (32.71, 33.53)	33.36 (32.83, 33.89)	0.24 (−0.44, 0.91)	0.49
Lateral tibial plateau temperature				
Week 2	33.54 (33.09, 33.99)	33.80 (33.46, 34.13)	0.26 (−0.30, 0.82)	0.37
Week 6	33.52 (33.15, 33.88)	33.59 (33.15, 34.02)	0.07 (−0.50, 0.64)	0.80
Medial femoral condyle temperature				
Week 2	33.19 (32.72, 33.66)	33.33 (32.86, 33.81)	0.14 (−0.52, 0.81)	0.68
Week 6	33.08 (32.68, 33.49)	33.29 (32.69, 33.89)	0.21 (−0.52, 0.93)	0.58
Lateral femoral condyle temperature				
Week 2	32.54 (31.99, 33.09)	32.66 (32.19, 33.13)	0.12 (−0.61, 0.84)	0.75
Week 6	32.43 (32.00, 32.85)	32.51 (31.96, 33.05)	0.08 (‐0.62, 0.78)	0.82
Supracondylar temperature				
Week 2	32.71 (32.17, 33.25)	33.07 (32.57, 33.58)	0.36 (−0.38, 1.10)	0.34
Week 6	32.74 (32.34, 33.15)	32.99 (32.40, 33.59)	0.25 (‐0.47, 0.97)	0.50
Traditional Chinese medicine syndrome grading and quantitative scoring				
Week 2	4.86 (4.35, 5.37)	6.14 (5.43, 6.86)	1.29 (0.41, 2.16)	0.004
Week 6	3.50 (2.96, 4.04)	5.21 (4.58, 5.85)	1.71 (0.88, 2.55)	< 0.001
PHQ‐9 health questionnaire				
Week 2	2.76 (1.67, 3.85)	4.31 (2.89, 5.73)	1.55 (−0.24, 3.34)	0.09
Week 6	2.10 (1.09, 3.10)	3.45 (2.27, 4.64)	1.36 (−0.20, 2.91)	0.09
Week 12	2.67 (1.61, 3.73)	3.40 (2.25, 4.56)	0.74 (−0.83, 2.30)	0.36

**FIGURE 3:**
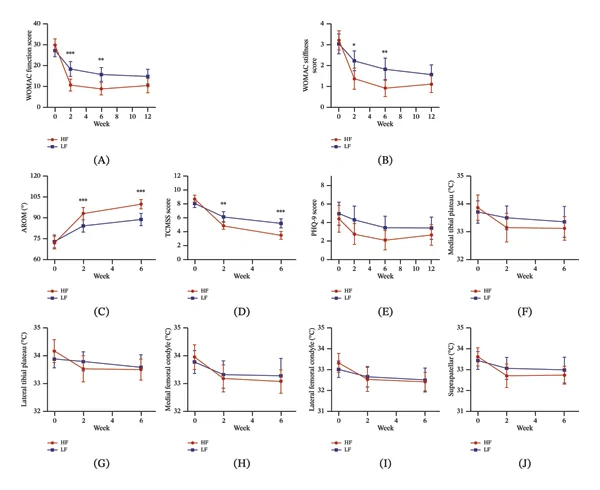
Trend chart of changes in all secondary outcome measures at different time points.

### 8.4. Adverse Events

Adverse events were graded using the Common Terminology Criteria for Adverse Events (CTCAE) Version 5.0. Throughout the 12‐week study period, adverse events were monitored at every treatment visit and at the week‐12 follow‐up. During the 6‐week treatment period, two mild (Grade 1) adverse events were reported, including one case in each group, corresponding to an incidence rate of 2.38% per group. Both events involved small localized skin blisters that resolved spontaneously without additional medical intervention. No serious adverse events or treatment discontinuations due to adverse events were reported. There was no statistically significant difference in adverse event incidence between the two groups (*χ* ^2^ = 0, *p* > 0.999).

## 9. Discussion

This randomized controlled study systematically compared the clinical efficacy and safety of warm acupuncture at different frequencies for treating KOA. The findings demonstrate that both high‐ and low‐frequency warm needle acupuncture significantly improved pain, joint function, and TCM syndrome manifestations. Importantly, the high‐frequency intervention consistently produced superior clinical outcomes across primary and most secondary endpoints, suggesting that treatment frequency is a critical determinant of therapeutic efficacy. This finding not only provides an optimized clinical application scheme for warm acupuncture in KOA treatment but also offers evidence‐based support for optimizing the frequency of nonpharmacological TCM interventions for osteoarthritis. The incidence of adverse events in both groups was only 2.38%, with no serious adverse events occurring throughout the trial. Compared to the adverse event rates reported in existing studies on conventional drug therapy for KOA (dry mouth 86.9%, constipation 58.6%, and sweating 31.3%) [16], warm acupuncture, as a TCM external therapy, directly targets local acupoints, thereby avoiding potential damage to gastrointestinal, hepatic, and renal functions associated with oral medications. This characteristic makes it more suitable for elderly KOA patients who are intolerant to drug therapy and aligns with the current clinical trend toward “green medicine.” This finding aligns with multiple domestic and international safety studies on acupuncture therapies for osteoarthritis [17], further validating the safety and reliability of warm acupuncture treatment.

High‐frequency warm acupuncture appeared to confer an early analgesic benefit at Week 2, as evidenced by a between‐group difference of 3.071 points in WOMAC pain scores. Several factors may account for this pronounced short‐term effect. First, the study enrolled patients with early‐to‐moderate KOA, a population characterized by substantial baseline pain variability and heightened responsiveness to short‐term acupuncture interventions. Second, the optimized high‐frequency warm acupuncture protocol employed in this study constitutes an intensified stimulation strategy, which may elicit rapid initial analgesic responses. Third, symptom fluctuations attributable to spontaneous regression, compounded by placebo and expectancy effects prevalent in early‐stage, mild‐symptom populations, likely contributed to the apparent magnitude of the short‐term effect. It is imperative to emphasize that the observed 2‐week symptomatic improvement should not be conflated with sustained long‐term efficacy, as it may partially reflect transient rather than durable clinical benefits. Furthermore, the early analgesic benefit observed at Week 2 persisted throughout the entire follow‐up period, and secondary outcome indicators including joint stiffness and physical function also demonstrated superior efficacy of high‐frequency warm acupuncture. This temporal trajectory aligns with findings from previous warm needle acupuncture studies in KOA, suggesting that early pain modulation may serve as a precursor to subsequent functional gains [18].

Selecting the ST35 and EX‐LE4 acupoints for warm needle acupuncture intervention regulates qi and blood circulation in the knee. This achieves the effects of unblocking meridians, promoting blood circulation, and resolving stasis, thereby alleviating muscle atrophy and pain and improving functional status in KOA patients [19]. Research confirms that warm acupuncture at ST35 and EX‐LE4 inhibits chondrocyte apoptosis by regulating the mRNA expression pathways of apoptosis‐regulating proteins caspase‐3 and caspase‐9 in cartilage tissue [20]. It also suppresses the NF‐κB signaling pathway to reduce inflammatory responses [21], thereby protecting articular cartilage and delaying disease progression. Acupuncture at points such as ST34, SP10, GB34, and SP9 effectively stimulates meridian energy flow, promotes knee blood circulation, and alleviates local inflammation, thereby reducing pain and improving muscle function [22]. Consistent, regular warm stimulation further enhances local knee blood circulation, promotes clearance of inflammatory mediators like IL‐1β, IL‐6, and TNF‐α, and reduces synovial inflammation and tissue edema [23]. Notably, no significant differences were observed between the two groups in PHQ‐9 total scores or knee joint temperatures. The PHQ‐9 scale focuses on psychological and emotional state assessment. While the 6‐week intervention period effectively improved physical symptoms, emotional state improvement may require a longer intervention period or combined targeted psychological counseling measures. This study found that knee‐related regional temperatures decreased in both groups posttreatment. This reduction is presumed to result from the effects of warm acupuncture on unblocking meridians and improving local circulation, thereby alleviating knee inflammation and restoring abnormal metabolic states. The lack of significant differences in skin surface temperature in the low‐frequency group may be attributed to insufficient cumulative treatment volume.

Although the precise mechanism of warm acupuncture in treating KOA remains incompletely understood, existing basic and clinical research allows reasonable inference regarding the efficacy differences between high‐frequency and low‐frequency interventions. As a treatment modality involving synergistic regulation of the “meridian‐nerve‐immune” system, the efficacy of warm acupuncture may be achieved through multitarget mechanisms: First, from an immunoregulatory perspective, both the mechanical stimulation of acupuncture and the thermal stimulation of moxibustion induce mast cell degranulation, releasing active substances such as histamine and serotonin. These substances stimulate adjacent nerve endings, mediating immune responses and pain regulation. High‐frequency warm acupuncture can sustain mast cell degranulation, continuously releasing active substances that act on adjacent nerve endings to regulate KOA pain [24].

Second, from the perspective of inflammation regulation, animal studies indicate that warm acupuncture can reduce the expression levels of inflammatory factors in synovial tissue by modulating the NF‐κB signaling pathway, thereby suppressing synovial inflammatory responses [25]. High‐frequency intervention may enhance this regulatory effect, leading to more pronounced alleviation of joint inflammation. Third, from the perspective of the local microenvironment, warm acupuncture downregulates microRNA‐27a expression in KOA rats, activating the phosphoinositide 3‐kinase/protein kinase B/mammalian target of the rapamycin signaling pathway. This inhibits excessive autophagy and apoptosis in chondrocytes, thereby delaying joint damage and degeneration [26]. High‐frequency stimulation may prolong and enhance the efficiency of soft tissue repair and functional recovery in KOA. Similar intervention frequency‐related studies have been reported in other TCM external therapies, providing strong corroboration for the conclusions of this study. Liu Cunzhi posits that acupuncture frequency relates to cumulative effects. Research indicates that electroacupuncture administered three times weekly achieves faster onset and more sustained therapeutic effects, making it suitable for acute pain or functional impairment in KOA. In contrast, weekly treatment requires eight weeks for cumulative effects; thus, weekly frequency is recommended for chronic pain management [27]. Guo Yan et al. observed differences in the efficacy of moxibustion needle therapy at varying frequencies for treating cold‐damp obstruction‐type KOA. They concluded that moxibustion needle therapy administered every 3 days demonstrated superior overall efficacy compared to treatment every 5 days. It more effectively reduced inflammatory factor levels, decreased joint effusion, and improved knee flexion and extension range of motion [28].

These studies collectively confirm that the frequency of TCM external therapy interventions is a key factor influencing efficacy, with high‐frequency interventions often yielding more substantial therapeutic effects. Furthermore, findings from physical therapy research indicating that high‐frequency rehabilitation training improves function in patients with musculoskeletal disorders [29] align with the core discovery of this study. This further supports the clinical understanding that “high‐frequency local interventions enhance treatment outcomes for musculoskeletal diseases,” providing interdisciplinary corroboration for optimizing the frequency of warm acupuncture.

However, this study has several limitations that require refinement in future research. First, blinding of participants and practitioners was not feasible due to the nature of the intervention, which may introduce performance bias. Additionally, the absence of a sham acupuncture control group precludes ruling out the influence of placebo effects on treatment outcomes. Future studies could design innovative sham stimulation control protocols, such as nonacupoint warm acupuncture or simulated acupuncture without thermal effects, to further clarify the specific therapeutic efficacy of warm acupuncture. Second, the high proportion of female participants (86.9%) aligns with the higher female prevalence of KOA in clinical settings [30]. However, this gender imbalance may limit the generalizability of findings to male populations. Future studies should optimize recruitment strategies to achieve more balanced gender distribution and enhance the universality of results. It should also be noted that participants with a history of previous acupuncture treatment were not excluded from this study. However, thorough baseline assessment and predefined sensitivity analyses confirmed that this did not affect the internal validity of the trial. Third, this study did not perform stratified analysis based on KOA severity, preventing clarification of the efficacy of differences of high‐frequency warm acupuncture across disease stages. Future stratified research targeting patients with varying disease severity is needed to provide more precise evidence for developing individualized clinical treatment plans. Fourth, the follow‐up period was relatively short, extending only to 6 weeks posttreatment, which may not fully capture the long‐term stability of moxibustion therapy effects. Future studies with extended follow‐up periods are needed to clarify the duration of efficacy for high‐frequency moxibustion. Fifth, in the present trial, most participants dropped out at an early stage of the intervention period. Although withdrawals were not primarily caused by unsatisfactory treatment response, outcome values at dropout were relatively poor. Accordingly, the LOCF approach in this setting is more likely to underestimate the true treatment effect. Finally, this study did not delve into the molecular biological mechanisms underlying how moxibustion frequency influences efficacy, such as specific regulatory effects on chondrocyte apoptosis or inflammatory cytokine expression. Future research integrating basic experimental studies could elucidate these mechanisms at the cellular and molecular levels, providing stronger theoretical support for the clinical application of moxibustion.

In summary, high‐frequency warm acupuncture demonstrates superior efficacy and safety compared to low‐frequency treatment for KOA, positioning it as a preferred nonpharmacological intervention. Future studies should address the limitations of this research by optimizing study design, extending follow‐up periods, and integrating basic experiments to further validate the efficacy and mechanisms of high‐frequency warm acupuncture, thereby providing more robust evidence for its clinical implementation in KOA management.

## 10. Conclusion

Both low‐ and high‐frequency warm acupuncture significantly improve clinical symptoms in patients with KOA. However, high‐frequency warm acupuncture demonstrates markedly superior efficacy compared to low‐frequency treatment in alleviating pain, enhancing joint function, increasing active knee range of motion, and improving TCM syndromes. As a TCM nonpharmacological therapy for KOA that combines both efficacy and safety, high‐frequency warm acupuncture provides reliable evidence‐based medical support for clinical nonpharmacological interventions in KOA management.

**Nomenclature**
KOA: Knee osteoarthritis
WOMAC: Western Ontario and McMaster Universities Osteoarthritis Index
PHQ‐9: Patient Health Questionnaire‐9
TCM: Traditional Chinese medicine
BMI: Body mass index
KL: Kellgren–Lawrence
AROM: Active range of motion
CTCAE: Common Terminology Criteria for Adverse Events

## Author Contributions

Yiqun Mi and Shifen Xu (principal investigators of the project): conceptualization, supervision, resources, and funding acquisition. Hanzhi Deng: conceptualization, methodology, data curation, and investigation. Qin Su: investigation and writing–review and editing. Yuqing Wang: data curation, project administration, and writing–original draft. Jiqing Wang, Kai Liu, and Liying Fu: patient treatment. Shanshan Li: statistical analysis and resources.

## Funding

This study was supported by the National Natural Science Foundation of China (Grant No. 82474619) and Shanghai Municipal Health Commission (ZY(2025‐2027)‐3‐2‐1).

## Disclosure

All authors reviewed and approved the final manuscript.

## Ethics Statement

This trial was conducted in accordance with the Declaration of Helsinki. Research ethics approval was obtained from the Ethics Review Committee of Shanghai Hospital of Traditional Chinese Medicine (2024SHL‐KY‐80‐02). Informed consent was obtained from each patient.

## Conflicts of Interest

The authors declare no conflicts of interest.

## Supporting Information

Additional supporting information can be found online in the Supporting Information section.

## Supporting information


**Supporting Information** Supporting Table 1 shows the acupoint selection, meridian attribution, and location used in the study. Supporting Table 2 presents detailed information including the specific therapeutic method, needle type, needle sensation, moxa cone type, frequency, and duration. Supporting Table 3 displays the assessment time points of all outcome measures in the study. Supporting Figure 1 shows the graphical abstract of the study design, interventions, outcomes, and main findings. Supporting Figure 2 shows the schematic diagram of acupoint location.

## Data Availability

The data that support the findings of this study are available on request from the corresponding author. The data are not publicly available due to privacy or ethical restrictions.
