# Supplementary material for: Frequency‐Dependent Effects of Warm Needle Acupuncture in Patients With Knee Osteoarthritis With Cold‐Dampness Obstruction Syndrome: A Randomized Controlled Trial
Source: Pain Res Manag. 2026 Oct 7;2026:1674261. doi: 10.1155/prm/1674261 (PMC13643950; doi:10.1155/prm/1674261)
Supplement: Supplementary file 1 [file PRM-2026-1674261-s001.docx]

**eTable 1 Acupoint Locations and Their Corresponding Meridians**

| Acupoints | Meridian Attribution of Acupoints | Location |
| --- | --- | --- |
| Dubi (ST35) | Stomach Meridian of Foot-Yangming | On the knee, in the depression lateral to the patellar ligament, when the knee is flexed. |
| Neixiyan (EX-LE4) | Extra Meridian Points | On the knee, in the depression medial to the patellar ligament, when the knee is flexed. |
| Yanglingquan (GB34) | Gallbladder Meridian of Foot-Shaoyang | On the lateral side of the lower leg, in the depression anterior and inferior to the head of the fibula. |
| Yinlingquan (SP9) | Spleen Meridian of Foot-Taiyin | On the medial side of the lower leg, in the depression anterior and inferior to the medial condyle of the tibia. |
| Liangqiu (ST34) | Stomach Meridian of Foot-Yangming | On the anterior side of the thigh, 2 inches above the laterosuperior border of the patella, on the line connecting the anterior superior iliac spine and the lateral border of the patella. |
| Xuehai(SP10) | Spleen Meridian of Foot-Taiyin | On the medial side of the thigh, 2 inches above the mediosuperior border of the patella, when the knee is flexed. |
| Heding (EX-LE2) | Extra Meridian Points | On the knee, at the midpoint of the upper border of the patella. |

**eTable 2 Details about Interventions**

|  | **High-frequency group** | **Low-frequency group** |
| --- | --- | --- |
| **Acupoints** | ST35, EX-LE4, GB34, SP9, ST34, SP10, EX-LE2 | Same as the high-frequency group. |
| **Acupuncture Operation Method** | For EX-LE4 and ST35, insert the needle obliquely upward to a depth of 25-40 mm; for the other selected acupoints, insert the needle perpendicularly to a depth of 20-30 mm. | Same as the high-frequency group. |
| **Needle type** | Acupuncture needles were selected from Wuxi Jiajian Acupuncture Needles with a specification of 0.3 mm×40 mm. | Same as the high-frequency group. |
| **Needle sensation** | The even reinforcing-reducing manipulation was adopted, and the needles were retained after deqi was achieved. | Same as the high-frequency group. |
| **Moxa Cone Type** | Moxa cones were selected from Nanyang Ya Yibao Moxa Products Co., Ltd. with a specification of 15 mm×10 mm. | Same as the high-frequency group. |
|  |  |  |
| **Moxa Cone Operation Method** | Place the moxa cones on the needle handles of ST35 and EX-LE4, controlling the distance between the moxa cones and the skin to be approximately 1.5 cm. Ignite the moxa cones from the bottom for moxibustion, with each moxa cone burning for 10 minutes, and burn 2 moxa cones continuously. | Same as the high-frequency group. |
|  |  |  |
| **Frequency and duration** | Treatment period: once a day, five times a week with two consecutive rest days after five days of continuous treatment. | Treatment period: once a day, twice a week with an interval of three days between each treatment session. |
|  | Maintenance period: once a day, once a week. | Same as the high-frequency group. |

**eTable 3. Time nodes of the study design**

| Timepoint | Enrollment Week -1 | Allocation Week 0 | Treatment | | Follow-up |
| --- | --- | --- | --- | --- | --- |
|  |  |  | Week 2 | Week 6 | Week 12 |
| Enrollment | × |  |  |  |  |
| Informed consent | × |  |  |  |  |
| Baseline assessments |  | × |  |  |  |
| Randomization |  | × |  |  |  |
| Intervention |  |  | × | × |  |
| Primary outcomes |  |  |  |  |  |
| WOMAC Stiffness Score |  | × | × | × | × |
| Secondary outcomes |  |  |  |  |  |
| WOMAC Stiffness Score |  | × | × | × | × |
| WOMAC Function Score |  | × | × | × | × |
| Active Range of Motion of the Knee Joint |  | × | × | × |  |
| Medical Infrared Thermography |  | × | × | × |  |
| Graded Quantitative Scale for TCM Syndrome Differentiation |  | × | × | × |  |
| PHQ-9 Health Questionnaire |  | × | × | × | × |
| Adverse events monitoring |  |  | × | × | × |

Note: Adverse events were assessed at every treatment visit and at the week-12 follow-up visit. Data represent aggregate adverse events at each assessment time point.

**eFigure 1.** **Graphical Abstract**

**eFigure 2. Locations of Acupoints**

(The picture was authorized by <https://www.figdraw.com)>
